# Cell Sheet Comprised of Mesenchymal Stromal Cells Overexpressing Stem Cell Factor Promotes Epicardium Activation and Heart Function Improvement in a Rat Model of Myocardium Infarction

**DOI:** 10.3390/ijms21249603

**Published:** 2020-12-16

**Authors:** Konstantin V. Dergilev, Evgeny K. Shevchenko, Zoya I. Tsokolaeva, Irina B. Beloglazova, Ekaterina S. Zubkova, Maria A. Boldyreva, Mikhail Yu. Menshikov, Elizaveta I. Ratner, Dmitry Penkov, Yelena V. Parfyonova

**Affiliations:** 1 National Medical Research Center of Cardiology, Russian Ministry of Health, Moscow 121552, Russia; doctorkote@gmail.com (K.V.D.); tsokolaevazoya@mail.ru (Z.I.T.); irene.beloglazova@gmail.com (I.B.B.); ver-mishel@mail.ru (E.S.Z.); mboldyreva@inbox.ru (M.A.B.); myumensh@mail.ru (M.Y.M.); eiratner@gmail.com (E.I.R.); Dpenkov@yahoo.com (D.P.); yeparfyon@mail.ru (Y.V.P.); 2 Federal Center of Brain Research and Neurotechnologies, Federal Medical Biological Agency, Moscow 117997, Russia; 3 Research Institute of General Reanimatology, Russian Academy of Medical Sciences, Moscow 107031, Russia; 4 Faculty of Medicine, Lomonosov Moscow State University, Moscow 119991, Russia

**Keywords:** stem cell factor, adipose derived mesenchymal stromal cells, cell sheet, myocardial infarction, heart function

## Abstract

Cell therapy of the post-infarcted myocardium is still far from clinical use. Poor survival of transplanted cells, insufficient regeneration, and replacement of the damaged tissue limit the potential of currently available cell-based techniques. In this study, we generated a multilayered construct from adipose-derived mesenchymal stromal cells (MSCs) modified to secrete stem cell factor, SCF. In a rat model of myocardium infarction, we show that transplantation of SCF producing cell sheet induced activation of the epicardium and promoted the accumulation of c-kit positive cells in ischemic muscle. Morphometry showed the reduction of infarct size (16%) and a left ventricle expansion index (0.12) in the treatment group compared to controls (24–28%; 0.17–0.32). The ratio of viable myocardium was more than 1.5-fold higher, reaching 49% compared to the control (28%) or unmodified cell sheet group (30%). Finally, by day 30 after myocardium infarction, SCF-producing cell sheet transplantation increased left ventricle ejection fraction from 37% in the control sham-operated group to 53%. Our results suggest that, combining the genetic modification of MSCs and their assembly into a multilayered construct, we can provide prolonged pleiotropic effects to the damaged heart, induce endogenous regenerative processes, and improve cardiac function.

## 1. Introduction

Since the 2000s, the cell-based therapeutic strategy has been developing as a promising option for treating cardiovascular disease, particularly myocardium infarction (MI). Conventional interventions cannot resolve the problem of extensive irreversible loss of cardiomyocytes and tissue scarification while organ transplantation is still accessible only for a miniscule proportion of patients. A paradigm of using cells to promote the regeneration and replacement of damaged tissue was initially very attractive, but raised an ongoing debate with perspective over time. Despite promising results in preclinical studies, this approach is still far from full-scale implementation mostly due to clinical trials data that fail to support it thus far [1,2,3].

While the potential use of embryonic or induced pluripotent stem cells is related to safety concerns regarding teratogenicity and oncology risk, different adult progenitor cell types like bone marrow cells, mesenchymal stromal cells (MSCs), skeletal myoblasts, endothelial precursors, and cardiac stem cells showed significant therapeutic effects in animal studies [3,4,5,6,7,8,9]. However, expectations that cell-based therapeutics will possess high myogenic potential and will directly replace the damaged tissue have not been fulfilled. Nowadays, it is believed that the paracrine activity of transplanted cells underlies their effects in animals. Still, some scientists are doubtful here, again proposing that cell injections just provide the induction of local immunity [10]. As for cardiac progenitors, the significance and magnitude of their regenerative capacity is under continuing debate [11,12,13,14].

High expectations are relevant to MSCs known for their immunomodulatory, clonogenic potential, secretory activity, easy access and propagation [15,16]. Still, the use of MSCs in a number of MI clinical studies showed mostly moderate and short-term effects. It is believed that the major challenges that cell therapy faces today are poor survival and engraftment of transplanted cells, their damage and loss through intracoronary/intramuscular injections and under conditions present in post-infarcted microenvironment, and, thus, insufficient efficacy [14]. Along with the efforts to define an optimal cell type(s), dose, time and route of administration, new strategies are being developed including but not limited to genetic modification, scaffold technologies, the use of multilayered cell constructs or cell-derived products like conditioned medium, exosomes, microRNAs [17].

In this regard, considerable attention is paid to genetic modifications of MSCs to enhance their paracrine capacity, improve their homing, retention, and survival [18]. Thus, an apoptotic caspase 8 knockdown in MSCs [19] or introduction of Akt1 protein kinase [20] reduced transplanted cell death and elimination. MSCs that overexpressed bone morphogenetic protein (BMP-2) promoted bone repair [21]. MSCs modified to secrete neurotrophins, like, for example, glial cell-derived neurotrophic factor (GNDF) promoted axonal growth, nerve repair and remyelination in nervous system damage models [18]. The effects were significantly higher when compared to unmodified MSCs. In our own studies, we showed that modification of MSCs by adeno-associated virus encoding VEGF promoted angiogenesis and tissue repair in a mice hind limb ischemia model [22]. In another study, we demonstrated neuroprotective potential of conditioned medium from adipose derived MSCs, overexpressing hepatocyte growth factor (HGF), that increased neurite outgrowth and glial cell migration in the dorsal root ganglion explant model. Upon transplantation, these cells stimulated nerve growth in ischemic skeletal muscle [23].

In this study, we hypothesized that therapeutic potential of MSCs for cardiac repair can be augmented by modifying them to secrete high level of stem cell factor (SCF). SCF is a known biologically active molecule, a ligand of c-kit receptor. It promotes the proliferation, survival, and migration of c-kit–expressing hematopoietic stem cells, endothelial progenitors, and cardiac stem cells [24]. Will the local production of SCF by transplanted MSC promote the mobilization of cardiac progenitors in the damaged tissue and improve cardiac repair and function? To test this, we generated gene-modified adipose–derived SCF-MSCs and evaluated their potential in a rat MI model. We utilized cell sheet (CS) technology to increase the cells survival and therapeutic potential. The idea was to apply multi-factorial stimuli to the damaged heart by combining the paracrine activity of MSCs with a pleiotropic effect of SCF.

## 2. Results

### 2.1. Characteristics of Cell Sheets Comprised of SCF-Producing MSCs

**Figure 1:**
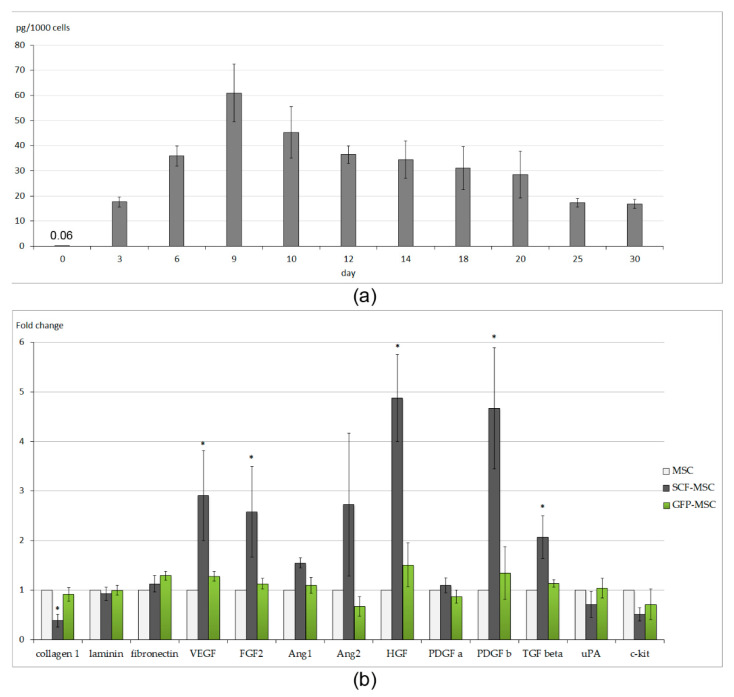
Paracrine and expression activity of SCF-MSC. (**a**) The dynamics of SCF protein level in the conditioned media from SCF-MSC evaluated by immunosorbent assay. The “day 0” bar indicates the basal level of SCF secretion by unmodified MSCs; (**b**) Expression profile (mRNA level) of SCF-MSC compared to unmodified cells or MSCs that were transduced by green fluorescent protein (GFP)-encoding AAV. The black and white bars indicate MSC-SCF CS and MSC CS groups, respectively. Data are presented as mean ± SD; *—vs. control MSCs, *p* < 0.05.

Rat adipose-derived MSCs were transduced with the recombinant adeno-associated virus (AAV) encoding the rat SCF gene. The AAV-DJ serotype used was previously shown to provide up to 80–90% efficacy of rodent MSCs transduction [23]. The level of SCF secretion by generated SCF-MSCs, evaluated by immunosorbent assay, peaked at day 9 post viral transduction reaching 60.97 ± 11.48 pg/mL (adjusted per 1 × 10^3^ cells). Transgene production retained for up to 1 month, though it went down to 15.53 ± 1.84 pg/mL by day 30 (Figure 1a). Even at day 3 it was almost 300-fold higher (17.64 ± 1.95 pg/mL per 1 × 10^3^ cells) than the basal level of SCF production by unmodified MSC (0.06 ± 0.02 pg/mL per 1 × 10^3^ cells).

We found a decrease in SCF-MSCs proliferative activity. Analyzing MSC distribution by cell cycle stages we observed a slight increase in the number of cells committed to S phase (less than 3% of population), while the number of cells in G2/M phase decreased (Supplementary Figure S1). This observation was consistent with previously published data [22,25] indicating the reduction of the primary cell cultures proliferation via growth arrest at the S or G2/M phases [26,27] following AAV infection.

We next utilized the SCF-producing MSC or unmodified cells to generate multilayered cell sheet constructs (SCF-MSC CS and MSC CS groups, respectively). Upon the detachment, the cell sheets underwent retraction resulting in a round-shaped patch (Supplementary Figure S2) with a diameter from 1.24 ± 0.05 cm (MSC CS, *n* = 8) to 1.68 ± 0.06 cm (SCF-MSC CS, *n* = 4). The diameter of cell sheets comprised of green fluorescent protein (GFP) expressing MSCs was 1.25 ± 0.09 (*n* = 7). Since an equal number of MSC or SCF-MSC was used to generate a cell sheet, this variety in an averaged size patch may be explained by variations in extracellular matrix (EM) content. Histological analysis showed extensive EM production (staining for collagen type I, fibronectin) both in SCF-MSC CS and MSC CS (Supplementary Figure S1). At the same time, the expression level (evaluated by quantitative real-time polymerase chain reaction-PCR) of collagen type 1 was reduced in SCF-MSC CS (Figure 1b) probably owing to decrease in density. An expression of other extracellular matrix proteins (laminin, fibronectin) did not vary between groups. In compliance with our previous findings [22], obtained CSs were characterized by low proliferation and apoptosis rate (singular events of cells positive for Ki67 proliferation marker or caspase 3 apoptotic marker, respectively; data not shown).

The level of SCF production by SCF-MSC CS, comprised of 3 × 10^6^ cells, was high reaching the concentration of up to 100 ng/mL by day 2 following the construct formation. We were unable to evaluate the dynamics of transgene product secretion due to cell sheet self-detaches after 3 days in culture.

Taking into account that a subpopulation of adipose-derived MSCs express the c-kit receptor for SCF [28], we next examined whether genetic modification could have an effect on the paracrine activity of MSCs. Surprisingly, real-time PCR of SCF-MSC CS lysate showed overexpression of a variety of angiogenic factors (Figure 1b): vascular endothelial growth factor (VEGF), fibroblast growth factor-2 (FGF2), tumor growth factor-b (TGF-b), platelet derived growth factor-b (PDGF-b), and hepatocyte growth factor (HGF). The mechanism of such upregulation remains unclear. We could not find clear evidence for SCF-mediated transcriptional activation of VEGF, FGF2, TGF-b, PDGF-b, and HGF in published data. Thus, further experiments are needed to support this finding.

**Figure 2:**
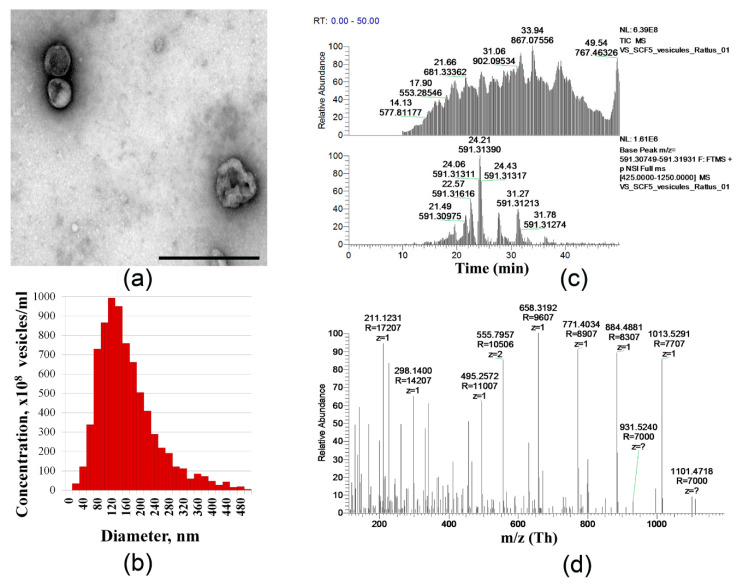
Paracrine and expression activity of SCF-MSC. (**a**) Representative transmission electron micrograph of EVs fractions, scale bar—500 nm; (**b**) A typical particle size distribution histogram of EVs from rat MSC obtained by Nanoparticle Tracking Analysis (NanoSight LM10); (**c**) Representative total ion current chromatogram (top) and extracted ion chromatogram (bottom) for SCF peptide (104–124 aa); (**d**) ESI-MS/MS spectrum of the SCF peptide (104–124 aa).

Accumulating data indicates an important role of extracellular vesicles (EVs) in the regulation of cell-to cell interaction, cell functions, and more importantly cardiac tissue protection [29,30]. We next evaluated the fraction of EVs that were secreted by SCF producing and unmodified MSCs. EVs were isolated using an ultracentrifugation method and characterized by the transmission electron microscopy (TEM). We found that the amount of the vesicles harvested from equal numbers of unmodified MSC CSs or SCF-MSC CSs did not vary. TEM revealed characteristic structural organization of the vesicles (Figure 2a). The obtained EVs ranged from 90 to 500 nm (the mean particle size was 154 ± 11 nm) in diameter corresponding to exosomes and ectosomes (Figure 2b). Using electrospray ionization tandem mass spectrometry (ESI-MS/MS), we next analyzed the EVs protein content and did not find significant differences between MSC CS and SCF-MSC CS groups (less than 5% of unique proteins). The following proteins were identified exclusively in EVs from SCF-MSC CS: peroxyredoxins Prdx 5, Prdx6, and superoxide dismutase SOD2, 60S acidic ribosomal protein P1, collagen V α-1, collagen type XIV α-1 chain, procollagen-lysine, 2-oxoglutarate 5-dioxygenase 1, nucleolin, serine hydroxymethyltransferase. More importantly, the analysis showed the presence of SCF peptide fragments (Figure 2c,d) in the spectra of EVs from SCF-MSC CS (not found in the vesicles from unmodified or green fluorescent protein (GFP)-expressing MSCs). Overall, extracellular vesicle protein cargo was presented the proteins linked to metabolic processes, cytoskeleton, and chaperones. More detailed information can be found in Supplementary Figure S3 representing the heat map proteomics data.

### 2.2. SCF-MSC CS Transplantation Prevents Tissue Fibrosis, Stimulates Amplification of C-Kit and WT1 Positive Cells in Post-Infarcted Myocardium and Improves Left Ventricle Function

We next conducted a set of experiments in a rat MI model to assess the viability of transplanted cells after their epicardial implantation in the form of CS, their integration/migration throughout the adjacent muscle tissue. Few minutes after ligation of the left coronary artery, pre-labeled (using PKH 26 fluorescent dye) SCF-MSC CS or MSC CS were implanted onto epicardial surface of the infarcted myocardium. As mentioned above, we determined that the optimal CSs size could be achieved using 6-well culture plates. That resulted in 1.2 + 0.4 cm (in diameter) patches which could cover both the infarction area and the peri-infarction zone (totally more than 0.6–0.7 cm in diameter).

**Figure 3:**
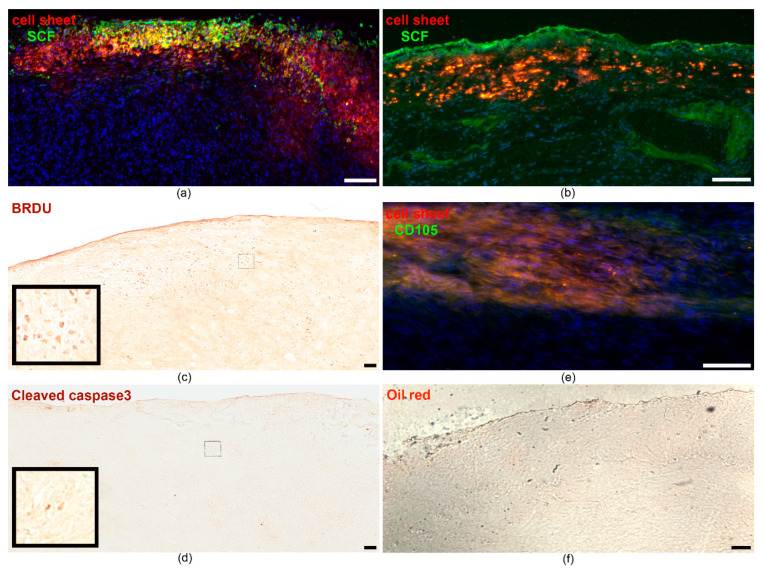
SCF-MSC CS integration into myocardium tissue and transgene expression at day 5 (**a**) and 14 (**b**) after MI induction. Representative images of the infarction area that is covered by the cell sheet comprised of PKH26-labeled cells (red). Specific staining indicates SCF protein (green) distribution throughout the muscle tissue; (**c**) Bromodeoxyuridine (BRDU) immunohistostaining to identify proliferating cells in the graft and adjacent tissue (day 14). The small square bar indicate the area of the section where the magnified view (large square bar) was taken from; (**d**) Immunohistostaining for cleaved caspase 3 to identify apoptotic cells in the graft and adjacent tissue (day 14). The small square bar indicate the area of the section where the magnified view (large square bar) was taken from; (**e**) Immunofluorescent staining for CD105 (green) at day 14; (**f**) Oil-red staining to identify adipose cells (day 14). Nuclei are stained with DAPI (4′, 6-diamidino-2-phenylindole). Scale bar-100 µm.

At days 5 and 14 after transplantation of CSs, the hearts were excised and subjected to histological analysis to assess the fate of the grafts in acute and early phase of tissue damage and inflammation. An integration of the cell graft into the underlying myocardium and the migration of labeled cells into the surrounding tissue was observed. Importantly, transplanted cells retained SCF production, which was evident by specific immunofluorescent staining (Figure 3a,b). By day 14, the graft appeared to be covered by the epicardial layer, integrated into the cardiac wall and lost structural integrity (Figure 3b). The latter could be due to both transplanted cell distribution throughout the adjacent tissue and to the infiltration of the graft by immunocompetent cells. Staining for bromodeoxyuridine identified proliferating cells within the cell sheet (Figure 3c), while only single apoptotic events (cleaved caspase 3 positive cells) were detected in the graft (Figure 3d). Along with continued transgene expression, this confirmed the viability of administered MSCs. In order to assess the risk of spontaneous differentiation of adipose-derived MSCs, specific oil-red staining was performed. No signs of adipocytes were found (Figure 3f), while the cells in the graft expressed mesenchymal stromal cell marker, CD105 (Figure 3e).

**Figure 4:**
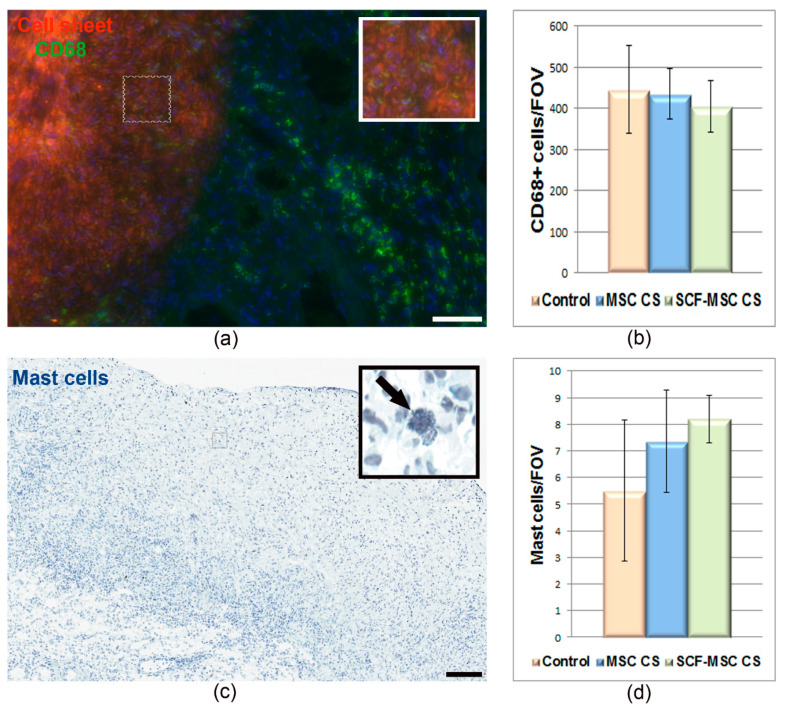
Macrophages and mast cells count at day 5 following MI induction and CS transplantation. (**a**) A representative image of myocardium section stained against CD68 (green), a marker of macrophages/monocytes. Nuclei are stained with DAPI. Scale bar-100 µm. The small square bar indicate the area of the section where the magnified view (large square bar) was taken from; (**b**) CD68+ cell count in the sham-operated, MSC CS and SCF-MSC CS groups. Data are presented as mean ± SD (Mann–Whitney U-test); (**c**) A representative image of myocardium section stained with toluidine blue to identify the mast cells. Scale bar-100 µm. The small square bars indicate the area of the section where the magnified view (large square bars) was taken from; (**d**) Mast cells count in the sham-operated, MSC CS and SCF-MSC CS groups. Data are presented as mean ± SD (Mann–Whitney U-test).

We next partially evaluated an immune response at the early stage (day 5) after MI induction and graft transplantation. Immunofluorescent staining against macrophages marker CD68 showed wide distribution of immune cells, both throughout the cell sheet and the adjacent myocardium (Figure 4a). At the same time, no difference in CD68+ cells count was found between groups (Figure 4b). A relatively small number of mast cells was detected in the graft and surrounding tissue (Figure 4c,d). Surprisingly, while the mast cell express c-kit receptor, the SCF-MSC CS group did not vary from controls (Figure 4d).

**Figure 5:**
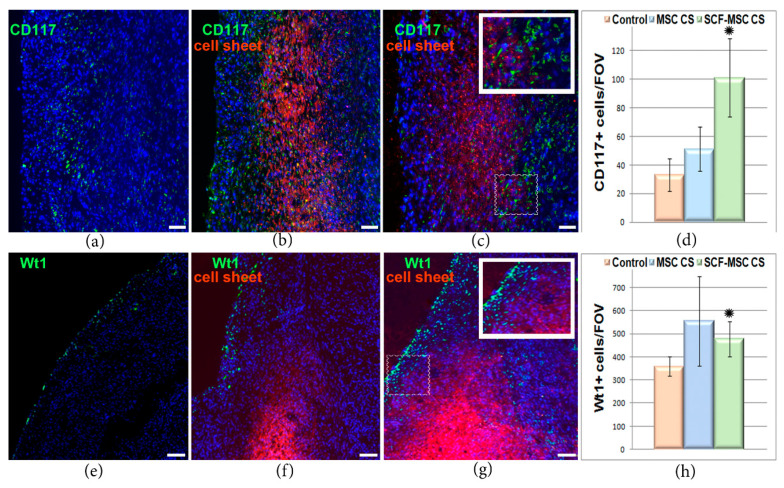
Histological analysis of the ischemic myocardium at day 5 after MI induction and CSs transplantation. (**a**–**c**) Representative images of myocardium sections stained for Wt1+ cells (green) from the sham-operated group (**a**), MSC CS (**b**) and SCF-MSC CS (**c**) group. PKH26-labeled MSCs comprising the cell sheet are in red. Nuclei are stained with DAPI. Scale bar-100 µm. The small square bar indicate the area of the section where the magnified view (large square bar) was taken from; (**d**) Wt+ cells count in the epicardial area of the section; data are presented as mean ± SD (Mann–Whitney U-test), * -SCF-MSC CS vs. control group, *p* = 0.033. (**e**–**g**) Representative images of myocardium sections stained for c-kit+ cells (green) from the sham-operated group (**e**), MSC CS (**f**) and SCF-MSC CS (**g**) group. PKH26-labeled MSCs comprising the cell sheet are in red. Nuclei are stained with DAPI. Scale bar-100 µm. The small square bar indicate the area of the section where the magnified view (large square bar) was taken from; (**h**) c-kit+ (CD117+) cells count; data are presented as mean ± SD (Mann–Whitney U-test), * -SCF-MSC CS vs. control group, *p* = 0.0495.

Since transplanted CS produced high levels of SCF, we analyzed heart sections for the presence of cells that carry c-kit receptor. C-kit is a tyrosine kinase receptor for SCF known to be essential for proliferation, survival and migration of different cell types, including cardiac progenitors. At day 5 after MI induction and CS transplantation, we found an increased number of c-kit positive cells (Figure 5a–d) in ischemic myocardium from SCF-MSC CS group (101 ± 27 cells per field of view) which was almost 3 times higher than in control (33 ± 11 cell per field of view). This suggests an increased migration of c-kit+ cells into ischemic muscle, which can take part in tissue regeneration.

The utilized route of CS implantation can provide additional therapeutic effect by applying activation stimuli to epicardial cell populations. These cells undergo epithelial-to-mesenchymal transition to generate epicardium-derived progenitor cells known to play crucial role in neonatal organogenesis [31,32]. We analyzed the ischemic myocardium specimens and surprisingly found a robust increase in Wt1 positive cells in CS treated groups (Figure 5e–h). Wt1 is an early epicardial response gene which expression is upregulated in fetals in response to cardiac injury. The number of WT1+ cells was significantly higher (*p* = 0.033) in SCF-MSC CS group compared to sham-operated. Unfortunately, in spite of c-kit cell accumulation and epicardium activation we did not observe any meaningful signs for cardiomyocytes lineage differentiation (staining for cardiac muscle-specific alpha-myosin heavy chain, αMHC; data not shown) 5 or 14 days following SCF-producing MSC sheet implantation.

**Figure 6:**
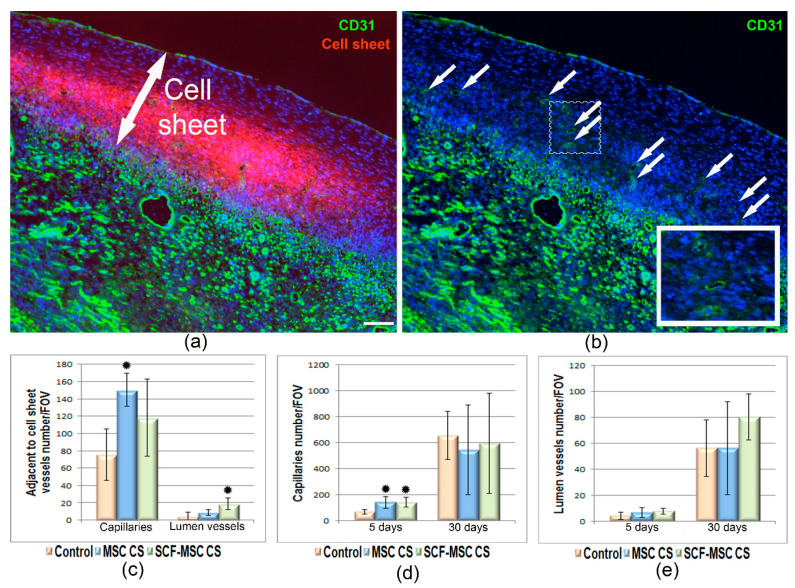
Vascularization of ischemic myocardium at day 5 and 14 after MSC sheet transplantation. (**a**,**b**) Representative images of CD31 positive vascular structures (green) in ischemic myocardium covered by CS comprised of pre-labelled MSC (red). Day 14 after MI induction. Nuclei are stained with DAPI (blue). White arrows (**b**) indicate vascular structures present in the cell sheet. (**c**) Capillaries and lumen vessel count in FOVs covering the adjacent to CS region. The black asterisk (⁕) indicates statistical significance vs. control group; (**d**) Capillaries vessel count. The black asterisk (⁕) indicates statistical significance vs. control group; (**e**) Lumen vessel count. Data are presented as mean ± SD (Mann–Whitney U-test). Scale bar-100 µm.

Vascularization of infarcted myocardium was assessed at day 5 and 14 after surgery and CS transplantation by CD31 positive vessel count (Figure 6a,b). We found that at day 5 the number of capillaries (Figure 6d) was higher both in MSC CS (142 ± 44 per FOV) and SCF-MSC CS (141 ± 38 per FOV) groups compared to sham-operated animals (68 ± 17 per FOV). This difference leveled out by day 14 indicating the relatively high rate of physiological vascularization after injury. The number of lumen-containing vessels did not vary between groups at both time-points (Figure 6e). At the same time, the count restricted to those fields of view, that were immediately adjacent to the cell sheet, showed almost 2-fold increase in the density of lumen vessels in SCF-MSC CS group compared to controls (Figure 6c). CD31-positive staining was also observed throughout the transplanted cell sheets (Figure 6a,b). The predominant proportion of these structures were PKH negative indicating that new capillaries arised from host endothelial cells. Only single events for PKH + CD31 + cells were found suggesting possible endothelial differentiation of MSC or cell fusion.

### 2.3. SCF-MSC CS Transplantation Prevents Tissue Fibrosis and Improves Left Ventricle Function

**Figure 7:**
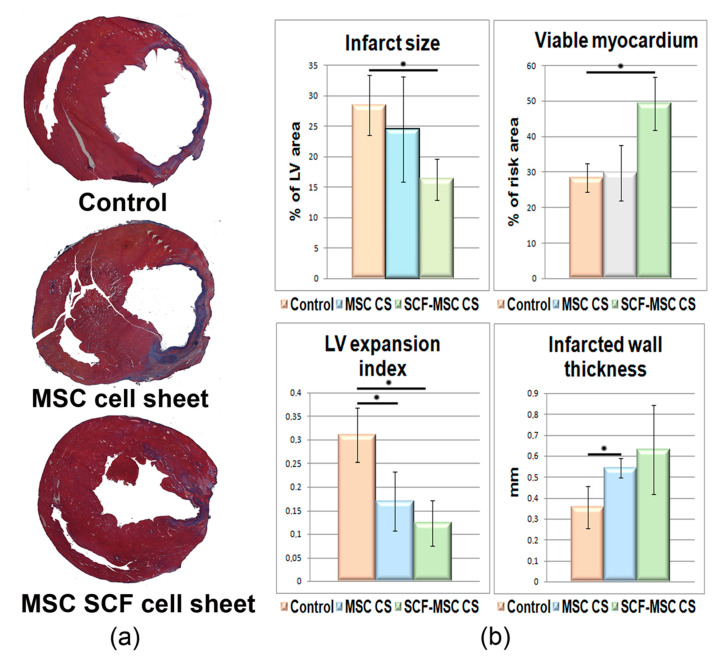
Morphometric analysis of LV remodeling at day 14 after CS transplantation. (**a**) Representative images of Mallory trichrome-stained myocardial sections from the sham-operated (control, *n* = 4), MSC CS (*n* = 5) and SCF-MSC (*n* = 6) groups: scar tissue and viable myocardium are in blue and red, respectively. (**b**) Quantitative analysis of LV morphometric parameters: infarct size, viable myocardium, infarcted wall thickness, LV expansion index. Data are presented as mean ± SD (Mann–Whitney U-test). The black asterisk (⁕) indicates statistical significance; infarct size: MSC SCF CS vs. control group, *p* = 0.02; viable myocardium: MSC SCF CS vs. control group, *p* = 0.02; LV expansion: MSC SCF CS vs. control group, *p* = 0.04; infarcted wall thickness: MSC vs. control group, *p* = 0.025, MSC SCF vs. control group—not statistically significant.

We next evaluated the remodeling of the damaged heart. At day 14 after artery ligation and CSs transplantation, when the scar formation is already measurable we performed morphometry analysis which showed that both an infarct size (IS), a left ventricle expansion index (LVEI) were dramatically reduced (Figure 7) in SCF-MSC CS group (IS 16 ± 3%; LVEI 0.123 ± 0.049) compared to sham-operated (IS 28 ± 5%; LVEI 0.317 ± 0.06) and MSC CS groups (IS 24 ± 9%; LVEI 0.169 ± 0.062). Additionally, MSC treated groups showed decreased LV wall thinning contributing to lesser dilatation of LV cavity (Figure 7). Transplantation of cell sheets, that produce SCF, reduced fibrosis development (Figure 7). The ratio of viable myocardium (preserved islets of muscle cells surrounded by fibrotic tissue) was significantly higher in this group, reaching almost 49% compared to untreated (28%) or MSC CS group (30%).

Inspired by the morphometric data we performed an additional experiment to assess the effect of SCF-MSC CS transplantation on cardiac function. At days 7 and 30 after MI induction, rats were anesthetized and transthoracic echocardiography (TE) was performed (for pre-treatment data collection TE was also performed prior to coronary artery ligation) to assess the ejection fraction (EF) of the infarcted heart. It should be noted that while adopting the approach (MI induction and TE) we first accomplished TE at day 5 after MI modeling and, unfortunately, faced a problem of animal’s death during the procedure. We think that the period between surgery (under Zoletil anesthesia) and TE under the inhalation anesthesia was important and was too short in this case. We did not face that challenge when the day 7 time-point was taken.

**Figure 8:**
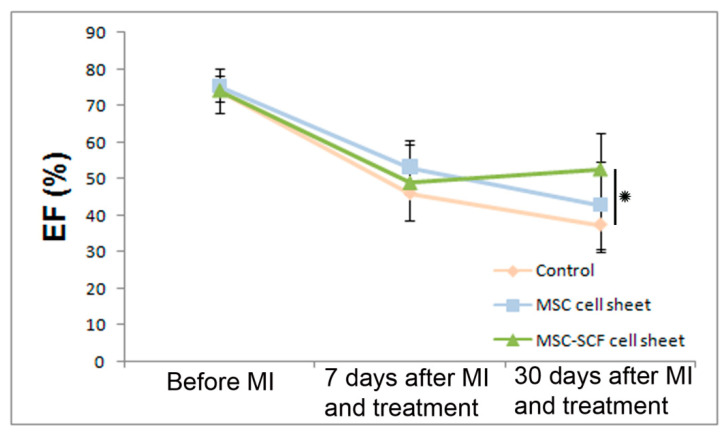
LV systolic function assessed by TE before and at different time-points (day 7 and 30) after MI and MSC CS (*n* = 10) or SCF-MSC CS (*n* = 10) transplantation. The black asterisk (⁕) indicates statistical significance; SCF-MSC CS vs. control group (*n* = 7), *p* = 0.037.

We found that the EF was moderately reduced in our MI model and had not dropped below 40% (Figure 8). No difference in systolic function was observed between groups by day 7. At day 30, the EF was significantly higher in SCF-MSC CS group (53%) versus sham-operated (37%) or MSC (43%) groups where.

## 3. Discussion

MSCs are considered the most promising candidates for cell-based cardiovascular therapy. They are easily accessible (especially from adipose tissue), possess high paracrine activity, and demonstrate a broad differentiation capacity. Importantly, MSCs hold potential to be used in allogeneic setting due to their immunomodulatory and immunosuppressive properties. MSC showed beneficial effects in thousands of preclinical studies. At the same time, completed clinical trials report their insufficient long-term efficacy. While a separate clinical study showed significant improvement in left ventricular functions after autologous MSCs transplantation [33], others [34,35] demonstrated only modest effects or non-significant tendency to cardiac remodeling and function improvement. Apart from the differences in clinical trial designs (cell type and source, dose, route of administration, inclusive criteria and readouts) this diversity between expectations and research outcomes is predominantly explained by the poor cell engraftment/survival following transplantation and the deficiency of damaged tissue replacement. Experimental data shows that up to 90% of cells dye in a few days after intracoronary injection [36]. An intravenous administration prevalent in animal studies often fails to provide cardiac homing of transplanted cells in humans. The survival of transplanted cells is also influenced by the inflammatory microenvironment in a post-infarcted myocardium under hypoxia, generation of free radicals and reactive oxygen species, apoptotic signals.

An effective way to improve the cell viability is the “cell sheet” approach. Within a multilayered construct, the cells retain their intercellular junctions, interaction with organized extracellular matrix and thus reside in close to native microenvironment. Our previous observations supported by other studies demonstrate the increased survival and therapeutic potential of CSs comprised of MSC in hind limb ischemia and peripheral nerve degeneration models [23,37,38,39]. To augment the therapeutic potential of MSCs and to confer more regenerative capacity we modified them to produce high levels of SCF. This biologically active factor [40] is known to play vital role in the regulation of different cells function and hemostasis. Our results demonstrate that cell sheets comprised of MSCs that overexpress SCF successfully integrate into infarcted myocardium after transplantation, retain viability and transgene production for relatively long period. More importantly, SCF producing CSs stimulated vascularization of ischemic muscle, prevented negative remodeling of cardiac tissue and led to improvement in cardiac function. These effects were superior to those described for unmodified cell sheets used. We suggest that the observed intense accumulation of c-kit+ cells in SCF-MSC CS treated animals may be a contribution to it. SCF by binding to its c-kit receptor promotes the activation of PI3K-AKT-MMP2/9, SDF-1α/CXCR4 signaling pathways, and modulates cell maintenance, differentiation, proliferation, and migration of cardiac progenitors [41,42,43,44,45]. Moreover, we found that modified MSC secrete extracellular vesicles that contain SCF. This looks intriguing since SCF was reported to interact with the intracellular form of the receptor and regulate endothelial cell differentiation [46]. A forming SCF gradient, thus, provides mitogenic and migration stimuli to ischemic myocardium and surrounding tissue and can promote activation of c-kit positive cardiac progenitor cells (CPC). These cells are capable of differentiation into cardiomyocites, smooth muscle, and endothelial cells. They produce different biological active molecules (angiotensin-1; hepatocyte growth factor, bFGF, insulin-like growth factor 1, PDGF, SCF, SDF-1, VEGF) and exert cardio-protective action [47,48]. Thus, the accumulation of CPC can contribute to the partial replacement of the lost cardiomyocytes and regeneration of the damaged tissue.

Importantly, we observed signs of epicardial cells activation. The epicardium plays an important role in promoting proliferation and survival of cardiomyocytes. Moreover, the epicardium alone is a valuable source of different cell types that take part in tissue protection and regeneration after injury [31,32,49]. Increased number of Wt1-positive cells was found in the epicardium and sub-epicardium from CSs treated animals. We suggest that the route of CS transplantation used in our study along with the high paracrine activity of transplanted cells promoted the epithelial–mesenchymal transition and the formation of epicardial progenitor cells. In this case, revealed upregulation of TGF -β and PDGF expression in SCF-MSCs is intriguing since these factors a known to be upstream regulators of epithelial-mesenchymal transition [31]. At this stage, the epicardium regains embryonic state and is characterized by the upregulation of Wt1 [50]. Epicardial progenitor cells migrate to the damaged tissue and differentiate into various cell types like myocardial cells and c-kit + telocytes, thus forming a cardiac “cell niches”. This interstitial network promotes cardiomyocyte regeneration after injury [51]. Thus, the amplification of WT1 positive cells can indicate the activation of a conserved epicardium-mediated cardiac repair mechanism following MSC derived cell sheet transplantation.

Undoubtedly, among limitations of the presented proof-of-concept study is a short-term cardiac function assessment period, only partially considered immune responses following SCF-MSC sheet transplantation, and insufficiently investigated SCF-mediated paracrine response of modified cells. These issues along with more in-depth histological analysis will be addressed in future studies.

In conclusion, the presented work demonstrates that the pleiotropic action of adipose-derived MSC sheet, that produces SCF, promotes accumulation of cardiac progenitors and Wt1-positive epicardial cells in post-infarcted myocardium, prevents negative remodeling, and finally improves cardiac function. These findings are important for understanding regeneration processes in the heart after injury and for the further development of cell therapy approaches to treat an aging human population that suffers from chronic cardiovascular disease.

## 4. Materials and Methods

### 4.1. Animals

Male Wistar rats (11–12-week-old) were purchased from “Pushchino” nursery for laboratory animals, Branch of Shemyakin–Ovchinnikov Institute of Bioorganic Chemistry, Russia. Animals were housed in individual cages at 22 °C and 12-h light/dark cycle. They were fed with standard chow and water ad libitum. Euthanasia was conducted under isoflurane narcosis by secondary cervical dislocation. Surgical manipulations and euthanasia procedures were developed in compliance with national and European Union directives and were approved by the Institutional Ethics Board for Animal Care (National Medical Research Center of Cardiology; permit #173, permission date 14.04.2017).

### 4.2. Cell Cultures

HEK-293T cell line was purchased from ATCC. Rat MSC were isolated from subcutaneous adipose tissue. Briefly, the tissue sample was fragmented under sterile conditions to a homogeneous mass and subjected to enzymatic treatment in Dulbecco’s modified Eagle’s medium (DMEM) medium (Gibco, Waltham, MA, USA) with 10 U/mL dispase (Worthington Biochemical, Lakewood, NJ, USA) and 200 U/mL type I collagenase (Worthington Biochemical, Lakewood, NJ, USA) at 37 °C for 30 min. After centrifugation for 10 min at 200× g the cell pellet was resuspended in DMEM and filtered through 40 μm nylon membrane. Isolated cells were maintained in 4.5 g/L D-glucose DMEM with 10% fetal bovine serum (FBS, Gibco, Waltham, MA, USA) and 1% penicillin/streptomycin solution at 37 °C and 5% CO_2_. For all experimental procedures, including CS generation and preliminary testing, early passage cells were used (P3–P4). The cells were passaged by Trypsin-Versene 1:1 solution (Paneco, Moscow, Russia).

### 4.3. Viral Vectors and Cell Transduction

Rat SCF coding sequence (NM_021843.4) flanked with BamH1 and BsrG1 restriction sites was synthesized and cloned into pAAV-MCS adeno-associated vector (Stratagene, San Diego, CA, USA) along with the “stuffer” BsrG1-Sal1 fragment from pX330-U6-Chimeric_BB-CBh-hSpCas9 (Addgene, Watertown, MA, USA) to generate pAAV-rSCF. Production of recombinant AAV particles was performed in HEK293T cells by cotransfection with pAAV-Dj (Cell Biolabs, San Diego, CA, USA), pHelper (Stratagene, San Diego, CA, USA) and vector plasmid pAAV-rSCF. To generate recombinant AAV encoding green fluorescent protein (GFP) pAAV–DJ, pHelper and pAAV–hrGFP (Stratagene, San Diego, CA, USA) were used. Cells that reached 80% confluency (8–10 × 10^6^ cells) were transfected using calcium-phosphate method and maintained for 48 h at 37 °C in DMEM supplemented with 10% FBS. Thereafter, the cells were detached and collected by centrifugation at 200× g for 10 min. Cell pellet was resuspended with 1 mL of PBS (per culture dish), subjected to four freeze–thaw cycles (liquid nitrogen/37 °C water bath) and then incubated with 25–50 U/mL of Benzonase (Merck, Darmstadt, Germany) at 37 °C for 30 min. Cell debris was removed by centrifugation at 5000× g (30 min) and the 1 mL of obtained viral stock was aliquoted and stored at −70 °C until use. Early-passaged (3–4) rat MSC were cultured on 100 mm culture dish until 70% confluency (corresponds to 0.8–1.0 × 10^6^ rat MSCs). The media was then replaced with 2 mL of DMEM + 3 mL of viral stock. The dish was agitated by tapping to ensure even cell distribution and incubated at 37 °C, 5% CO_2_ (tapping every 30 min). Then, 3 h after infection, 5 mL of DMEM containing 20% FBS and 1% penicillin/streptomycin solution were added. Transduced cells were cultured for 48 h prior to experiments.

To assess the distribution of MSCs by cell cycle stages, the cells were fixed in ice-cold 70% ethanol for 4 h. DNA was denaturized in 2 N HCl/0.5% Triton X-100 solution for 30 min, and then the suspension was neutralized by 0.1 M borate buffer (pH 8.5). The cells were resuspended in propidium iodide (50 µg/mL, Thermo Fisher Scientific, Waltham, MA, USA) and processed with BD FACS CantoTM II (BD Biosciences Pharmingen, San Diego, CA, USA). FCS Express 4 software (De Novo Software, Canada) was used to analyze the cell cycle distribution according to propidium iodide fluorescence intensity (exciting wave 561 nm, emission 610/620 nm).

### 4.4. Cell Sheet Formation

Prior to cell sheet formation MSC (passage 2–4) were labeled by PKH26 Red dye (PKH26 Red Fluorescent Cell Linker Kit for General Cell Membrane Labeling, Sigma-Aldrich, Milwaukee, WI, USA). The cells were detached with 0.05% trypsin, labeled and seeded at 3 × 106/well density in a 6-well culture plate (Corning, Corning, NY, USA). Forming CSs were maintained in DMEM, 10% FBS, penicillin/streptomycin for at least 48 h prior to experiments. The transgene expression was verified by enzyme-linked immunosorbent assay to assess SCF concentration in conditioned media. The Mouse SCF ELISA Kit (ab100740, Abcam, Cambridge, MA, USA) was used. An optical density was measured using VictorTM X3 Multi Label Plate Reader (Perkin Elmer Inc, Waltham, MA, USA). The cell sheet was harvested by incubation in Versene solution (Paneco, Moscow, Russia) until it self-detached within 5–7 min.

### 4.5. Real-Time PCR

Total RNA was isolated using RNeasy Mini Kit (Qiagen, Germantown, MD, USA). cDNA was obtained using RevertAidTM First Strand cDNA Synthesis Kit (Fermentas, Thermo Fisher Scientific, Waltham, MA, USA) and subjected to PCR in StepOnePlus™ Real-Time PCR System (Applied Biosystems, Thermo Fisher Scientific, Waltham, MA, USA) using specific primers listed in Table 1.

### 4.6. Extracellular Vesicles Isolation and Proteomics

Extracellular vesicles were isolated from MSC conditioned medium. The cells were washed with Hanks solution and maintained in DMEM/F-12 with 0.4% exosome-depleted FBS (System Biosciences, Palo Alto, CA, USA) for 48 h. The conditioned medium was subjected to gradient centrifugation (400× g for 10 min, 2000× g for 30 min) to sediment cells, cell debris, and aggregates of biopolymers. The supernatant was then centrifuged at 100,000× g for 90 min in a SW32 Ti baket rotor (Beckman Coulter, Brea, CA, USA) at 40 °C; Ultracentrifuge Optima XE-90 (Beckman Coulter, Brea, CA, USA). EV distribution by size and concentration was analyzed using NanoSight LM10 (NanoSight Ltd., Minton Park, UK). For TEM imaging EV were loaded on carbon-coated grids and negatively stained with 1% uranyl acetate solution. The grids were imaged at 80 kV using JEOL JEM-1011 transmission electron microscope (JEOL USA, Peabody, MA, USA) equipped with digital camera ORIUS SC1000W. The Shotgun mass spectrometry proteomics assay was used to analyze protein content of EV. A denaturing solution consisting of 5 M urea, 15% acetonitrile, 0.5% sodium deoxycholate, 300 mM sodium phosphate buffer (pH 6.0), and 5 mM trichloroethyl phosphate was added to EV. The mixture was treated in an ultrasonic bath (45 °C, 30 min), then a solution of 2% 4-vinylpyridine in 30% propan-2-ol was added. The alkylation reaction was carried out at 22 °C for 30 min. Then, trypsinolysis was performed: 2 h, 38 °C, at 1:50 (*w*/*w*) trypsin to substrate ratio and 2 h, 38 °C, at 1:100 (*w*/*w*). The reaction was stopped by adding the formic acid. Peptides were preliminary enriched on a C-18μ-PrecolumnPepmap (Thermo Scientific, Waltham, MA, USA) column and then separated on an analytical C-18 AcclaimPepmap (Thermo Scientific, Waltham, MA, USA) column in an acetonitrile gradient. Mass spectrometry was performed using high-resolution hybrid orbital mass spectrometer with a linear ion trap OrbitrapFusion/UltiMate 3000 Binaré RSLCnano HPLC system (Thermo Scientific, Waltham, MA, USA). A three-segment differentiated scanning method was used. Proteins were identified using Search Gui 3.3 software (CompOmics, Department of Biomolecular Medicine of the Faculty of Medicine and Health Sciences of Ghent University, Ghent, Belgium) by the X!Tandem Vengeance 12.15.2 search algorithm. Quantitative analysis was carried out based on the NSAF index (normalized spectral abundance factor) in a set of proteins common to a group of samples after alignment by the intensity of the reference spectra.

### 4.7. MI Modelling and CS Transplantation

MI was modeled in male 11–12 week-old Wistar rats as previously described [52]. Animals were anesthetized with Zoletil 100 (40 mg/kg, intraperitoneally, Virbac, Carros, France) and left thoracotomy was performed through the intercostal space. The pericardium was opened and the left anterior descending coronary artery was ligated. Rats were randomly assigned to the following three groups: (i) MSC CS, (ii) SCF-MSC CS, or (iii) sham-operated. Ten minutes after artery ligation the corresponding cell sheet was detached from the culture plate, untucked by sterile needle forceps and then transferred (using low-adhesive membrane) onto the epicardium layer (to cover the pale ischemic area). The implant was additionally fixated by 20 µL of “Tisseel” fibrin sealant (Baxter International, Deerfield, IL, USA). The heart was returned to the chest cavity, the muscles and skin were closed in layers. Control animals (sham-operated groups) underwent the same procedure except for cell sheet transplantation. To assess proliferation in ischemic myocardium at any time thereafter, 5-bromo-2’-deoxyuridine (BRDU) was administered by intraperitoneal injections (once a day, 100 mg/kg) for a period of 5 days.

At day 5 and 14 after MI induction, the rats were sacrificed. The hearts were excised and subjected to histological analysis. An echocardiography experiment was conducted at day 7 and 30 after MI induction and CSs transplantation.

### 4.8. Histological Analysis

Cell sheets, comprised of SCF-producing and unmodified MSC, were embedded in Tissue-Tek OCT compound (Sakura Finetek, Leiden, The Netherlands) and frozen in liquid nitrogen. Serial 7 μm-thick cross sections were prepared using Microm HM 505E (MICROM International GmbH, Walldorf, Germany), fixed in formalin and stained (1 h incubation) by primary antibodies against Ki-67 (Abcam, Cambridge, MA, USA) or cleaved caspase-3 (Cell Signaling Technology, Danvers, MA, USA) to analyze proliferation activity and apoptotic rate, respectively. To evaluate extracellular matrix content polyclonal antibodies for the collagen type I (Bio-Rad Laboratories, Hercules, CA, USA) and fibronectin (cat#6328-250, Abcam, Cambridge, MA, USA) were used. Following incubation with primary antibodies, the slides were stained with the corresponding secondary antibodies (Thermo Scientific, Waltham, MA, USA, 1/2000, 60 min, 37 °C).

For histological analysis, cardiac tissue sections were fixed in ice-cold acetone for 20 min, air-dried, and washed in PBS (5 min). The slides were then blocked by 10% normal donkey serum for 30 min followed by incubation with primary antibodies (diluted in a blocking solution-1% BSA in PBS): CD31 antibody (1:100, overnight; BD Biosciences Pharmingen, San Diego, CA, USA) to assess the capillary density (CD31-positive structures count per FOV); MHC antibody (Abcam, Cambridge, MA, USA, 1 h incubation) to evaluate cardiomyocyte differentiation. The slides were then washed in PBS and incubated with Alexa Fluor 488-conjugated secondary antibodies (Thermo Scientific, Waltham, MA, USA, 1:2000, 1 h at 37 °C). At the end of incubation, the nuclei were stained with DAPI. Microphotographs were taken in random fields of view covering the peri-infarction zone of the section.

For c-kit positive cells and activated epicardial cell count, sections were formalin-fixed and stained by primary antibodies against CD117 (Santa Cruz, USA, 1/50, + 4 °C overnight) and Wt1 (Abcam, Cambridge, MA, USA, 1/100, + 4 °C overnight), respectively. To assess the transgene expression antibodies against rat SCF (#PA5-20746, ThermoFisher Scientific, Waltham, MA, USA) were used. For mesenchymal stromal cell staining the formalin-fixed sections were incubated with primary CD105 antibodies (Abcam, Cambridge, MA, USA, 1/100, 1 h). Macrophages were stained with CD68 antibodies (Bio-Rad Laboratories, Hercules, CA, USA, 1/100, 1 h).

For mast cells count, the formalin-fixed sections were stained with toluidine blue for 60 min, washed by distilled water (three times), successively incubated in 70%, 96%, 100% ethanol (5 min in each solution), cleared in toluene and mounted.

To identify adipose cells staining with Oil Red O (Cat. # O-0625, Sigma-Aldrich, Milwaukee, WI, USA) was performed according to the following protocol: http://mousepheno.ucsd.edu/pdfs/1_Oil_Red_O_HC.pdf).

To identify proliferating cells (following 5-bromo-2’-deoxyuridine administration, Section 4.7), myocardium sections were stained with anti-BRDU antibodies (R&D Systems, Minneapolis, MN, USA, 1/100, 1 h) using ABC Elite kit (Vector Laboratories, Burlingame, CA, USA).

### 4.9. Morphometric Assay G-5562-2016

To assess cardiac tissue remodeling after MI and CS transplantation, animals underwent deep anesthesia (5% isoflurane inhalation), the hearts were arrested in diastole (by 0.1 mL saturated KCl injection into the left ventricular chamber) and excised. The atriums and large vessels were resected, while the left ventricle sample was washed with normal saline, embedded in Tissue-Tek OCT compound (Sakura Finetek, Leiden, The Netherlands) and frozen in liquid nitrogen. Serial 7 μm-thick cross sections were prepared (with 300 μm interval between sections) transversely from the apex to the base. Routine Mallory trichrome staining was performed; whole sections were photographed and then used for morphometry analysis using NIH ImageJ freeware. To assess the size of infarction, the ratio of the necrotic zone to the left ventricle area was determined. To measure the wall thickness, an average thickness of three equal segments was calculated. To quantitate both the degree of LV dilation and the infarction wall thinning, the LV expansion index was calculated using a modified method of Hochman and Choo [53]:

Expansion index = (LV cavity area/total area) × (non-infarcted region wall thickness/risk region wall thickness).

### 4.10. Echocardiography

Transthoracic echocardiography was performed by a blinded operator before (at baseline), and at day 7 and 30 post MI modeling and CS transplantation using Vevo-1100 echocardiography machine (VisualSonics, Amsterdam, The Netherlands). The rats were kept at 37 °C (on the heated plate) under 1.5% isoflurane inhalation during the procedure. The calculation of LV ejection fraction was obtained with 2-dimensional tracing.

### 4.11. Data Analysis

Values are presented as mean ± SD. Statsoft “Statistica 8.0” was used for data analysis. Statistically significant differences between the two groups were determined by a Mann–Whitney U-test depending on the sample distribution profile. Multiple groups were compared using ANOVA with Bonferroni correction where required. *p*-values less than 0.05 were considered indicative of significance.

## Figures and Tables

**Table 1:** Primers used for real-time PCR.

Gene	Primer Forward	Primer Reverse
B-Actin	AGCCATGTACGTAGCCATCC	CTCTCAGCTGTGGTGGTGAA
VEGF	ACTGGACCCTGGCTTTACTG	ACGCACTCCAGGGCTTCATC
Angiopoietin1	GTGGCTGGAAAAACTTGAGA	TGGATTTCAAGACGGGATGT
bFGF	AAGCGGCTCTACTGCAAG	AGCCAGACATTGGAAGAAACA
PDGFa	CCTGTGCCATCCGCAGGAAGAGA	TTGGCCACCTTGACGCTGCGGTG
PDGFb	GATCCGCTCCTTTGATGATC	GTCTCACACTTGCATGCCAG
HGF	CCAGCTAGAAACAAAGACTTGAAAGA	GAAATGTTTAAGATCTGTTTGCGTT
FGF2	ACGGCGTCCGGGAGAA	ACACTCCCTTGATGGACACAACT
TGF-β1	TGCTTCAGCTCCACAGAGAA	TGGTTGTAGAGGGCAAGGAC
c-kit	CACTCACGGGCGGATCAC	TCCGGTGCCATCCACTTCAC
Collagen I	CAACCTCAAGAAGTCCCTGC	AGGTGAATCGACTGTTGCCT
Fibronectin	CCTTAAGCCTTCTGCTCTGG	CGGCAAAAGAAAGCAGAACT
uPA	CTGACCCAGAGTGGAAAACAG	CGGCCATCGATGTTACAGAT

## Supplementary Materials

Supplementary materials can be found at https://www.mdpi.com/1422-0067/21/24/9603/s1. Figure S1: Characteristics of the cell sheets, comprised of adipose-derived MSC; Figure S2: Characteristics of the cell sheets, comprised of adipose-derived MSC; Figure S3: A proteomics heat map representing extracellular vesicles protein content.

## Abbreviations

MI	Myocardium infarction
MSCs	Mesenchymal stromal cells MSCs
SCF	Stem cell factor
CS	Cell sheet
AAV	Adeno-associated virus
EM	Extracellular matrix
VEGF	Vascular endothelial growth factor
FGF2	Fibroblast growth factor-2
TGF-b	Tumor growth factor-b
HGF	Hepatocyte growth factor
CPC	Cardiac progenitor cells
