# Supplementary figures and images for: Cell Sheet Comprised of Mesenchymal Stromal Cells Overexpressing Stem Cell Factor Promotes Epicardium Activation and Heart Function Improvement in a Rat Model of Myocardium Infarction
Source: Int J Mol Sci. 2020 Dec 16;21(24):9603. doi: 10.3390/ijms21249603 (PMC7766731; doi:10.3390/ijms21249603)

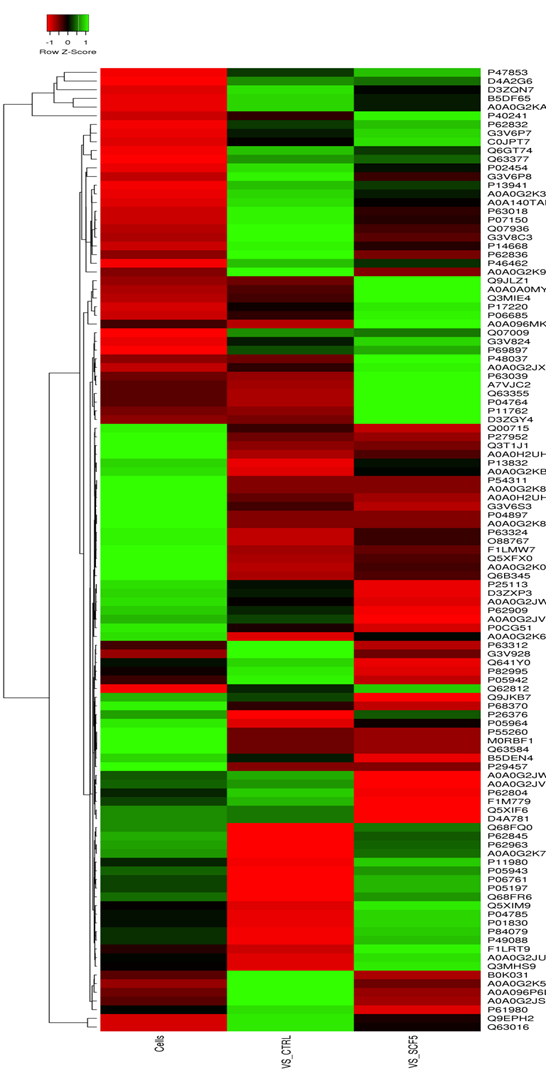

Supplement: Supplementary file 1 [file ijms-21-09603-s001.zip › Figure S3.tif]

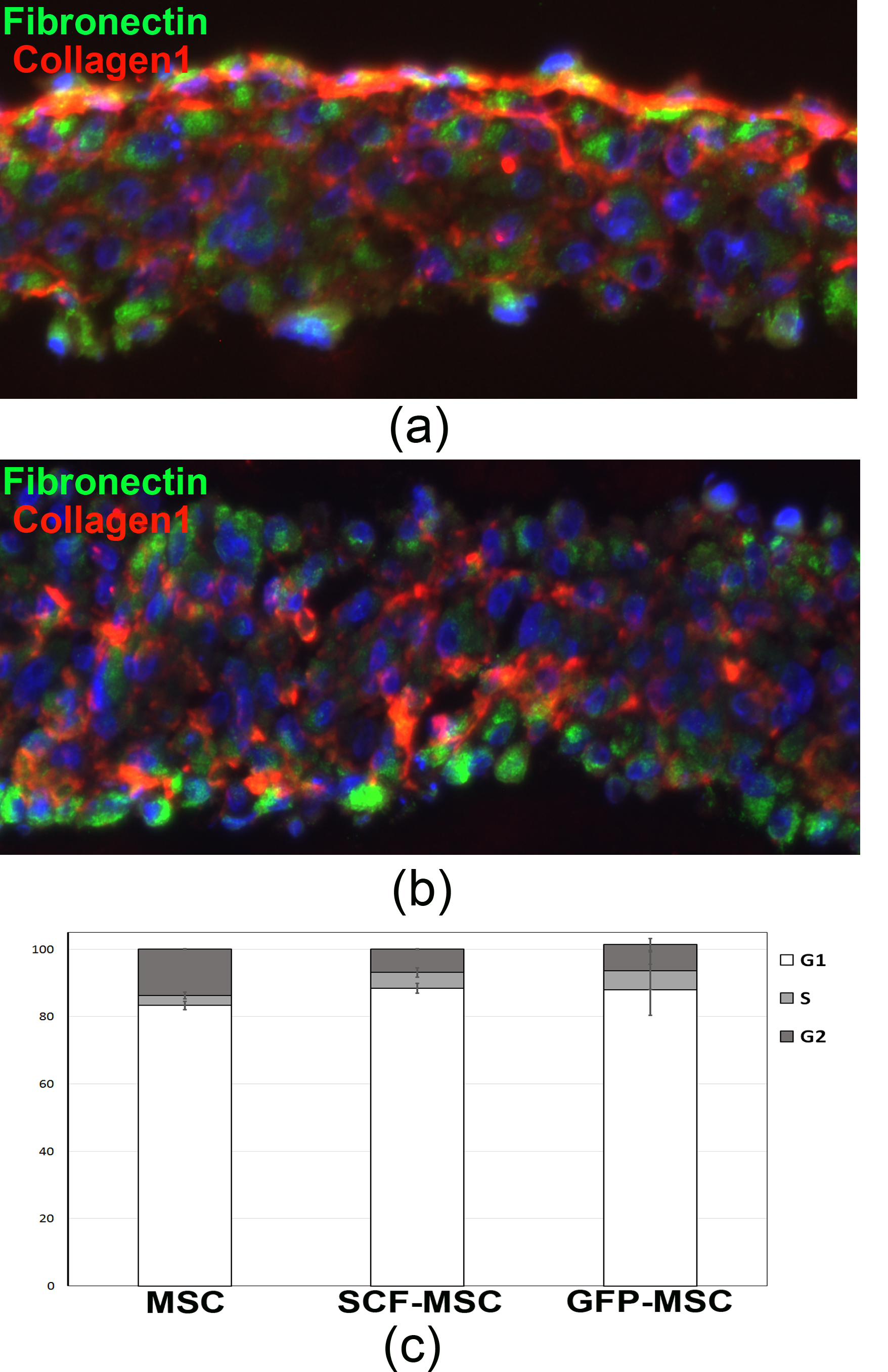

Supplement: Supplementary file 1 [file ijms-21-09603-s001.zip › Figure S1.tif]

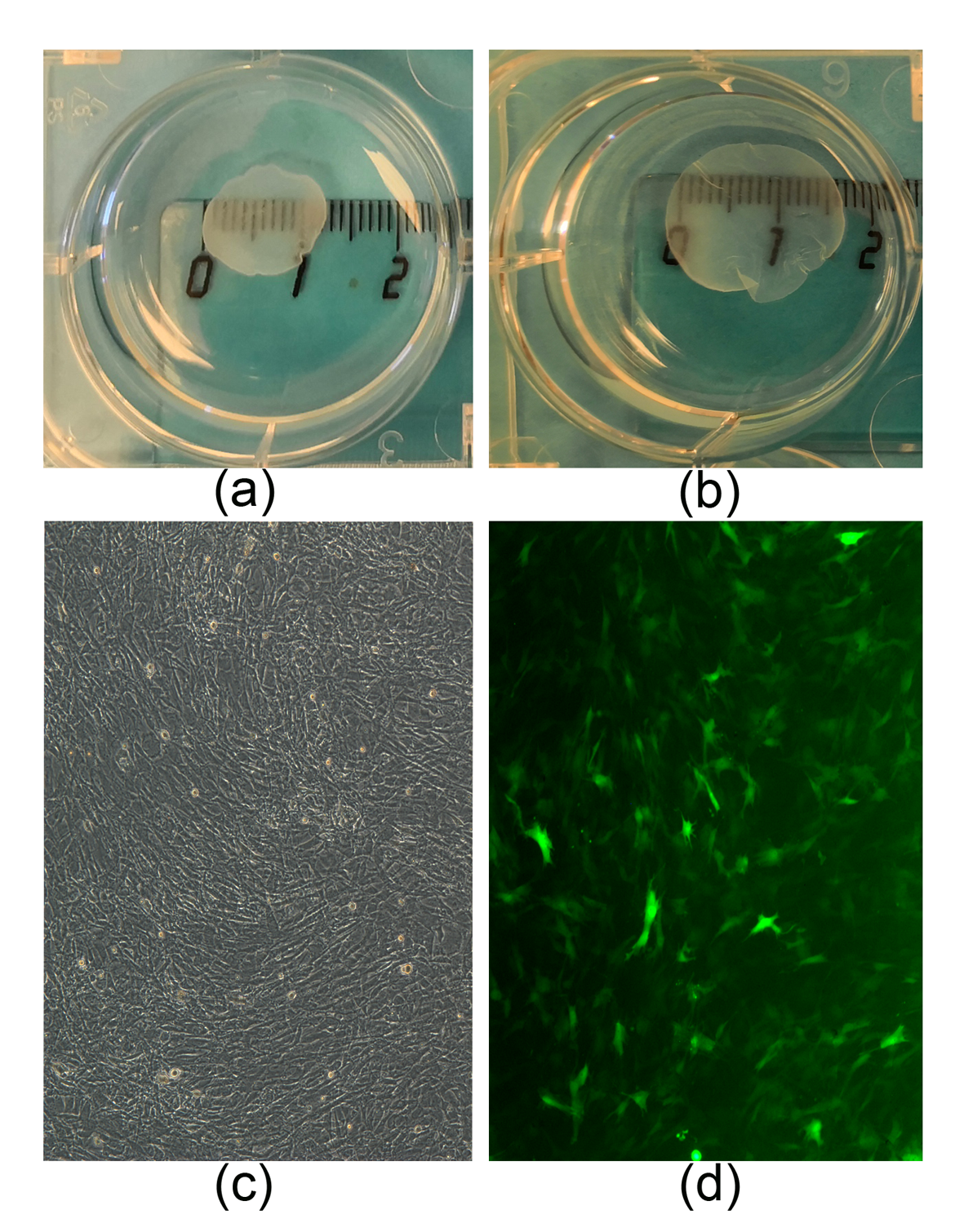

Supplement: Supplementary file 1 [file ijms-21-09603-s001.zip › Figure S2.tif]
